# Differential Pattern of Soluble Immune Markers in Asymptomatic Dengue, West Nile and Zika Virus Infections

**DOI:** 10.1038/s41598-019-53645-w

**Published:** 2019-11-20

**Authors:** Rafaelle Fares-Gusmao, Bruno Coelho Rocha, Emilia Sippert, Marion C. Lanteri, Germán Áñez, Maria Rios

**Affiliations:** 1 Office of Blood Research and Review (OBRR), Center for Biologics Evaluation and Research (CBER), U.S. Food and Drug Administration (FDA), Silver Spring, MD USA; 2 Blood Systems Research Institute, San Francisco, CA USA; 3 Department of Laboratory Medicine, University of California San Francisco, San Francisco, CA USA; 4 Present Address: Sanofi Pasteur, Swiftwater, PA USA

**Keywords:** Chemokines, Prognostic markers, Viral infection

## Abstract

Infections with dengue virus (DENV), West Nile virus (WNV) and Zika virus (ZIKV) usually present similar mild symptoms at early stages, and most infections (~80%) are asymptomatic. However, these infections may progress to severe disease with different clinical manifestations. In this study we attempted to identify unique characteristics for each infection at the presymptomatic/asymptomatic stage of infection and compared levels of soluble immune markers that have been shown to be altered during clinical course of these viral infections. Levels of soluble markers were determined by Luminex-based assays or by ELISA in plasma samples from asymptomatic blood donors who were reactive for RNA from DENV (n = 71), WNV (n = 52) or ZIKV (n = 44), and a control or non-infected (NI) group (n = 22). Results showed that even in the absence of symptoms, increased interleukin (IL) levels of IL-12, IL-17, IL-10, IL-5, CXCL9, E-Selectin and ST2/IL-1R4; and decreased levels of IL-13 and CD40 were found in all flavivirus group samples, compared to those from NI donors. DENV-infected donors demonstrated variation in expression of IL-1ra and IL-2; WNV-infected donors demonstrated variation in expression of IL-1ra, P-Selectin, IL-4 and IL-5; ZIKV-infected donors demonstrated variation in expression of IL-1ra, P-Selectin, IL-4, RANK-L, CD40L and C3a. The findings suggest that, even in the presymptomatic/asymptomatic phase of the infection, different immunomodulation profiles were associated with DENV, WNV and ZIKV infections.

## Introduction

Arthropod-borne viral (arbovirus) infections are among the most clinically relevant viral infections continually challenging public health systems in recent decades, mainly in developing countries. Dengue virus (DENV), West Nile virus (WNV) and Zika virus (ZIKV) are arboviruses from the genus *Flavivirus* (family *Flaviviridae*). They are transmitted by mosquitoes and cause endemic and epidemic disease around the world^1^.

Since its first reported epidemic in 1779-1780^2^, DENV has emerged and re-emerged worldwide, and is currently endemic in more than 100 countries^3^. Prediction studies indicate that approximately 390 million dengue cases occur around the world annually, of which 96 million are reported as severe disease^4^, with 500,000 people requiring hospitalization^3^.

WNV was first described in Uganda in 1937, and reported outside Africa since 1950, causing intermittent outbreaks in Europe and the Middle East. The virus reached the United States in 1999 as an emerging agent and spread quickly throughout the country, becoming endemic and causing recurring annual outbreaks for 21 years^5^. Between 1999 and 2017, 48,183 WNV cases were reported to Centers for Disease Control and Prevention (CDC), of which 2,163 resulted in death^6^.

ZIKV was first reported as infecting rhesus monkeys in Africa in 1947 and was later isolated from humans in Nigeria in 1968, with documented circulation in Africa until 1981^7^. It emerged outside Africa in 2007, causing an outbreak in Micronesia^8^, followed by outbreaks in French Polynesia (2013/2014)^9^, and Brazil (2015)^10^, among other countries. Since then, ZIKV has spread rapidly throughout the Americas, and has been estimated to infect more than one million individuals^11^. Associations between ZIKV infection and Guillain-Barré syndrome and microcephaly only were recognized after the 2015–2016 outbreak in Brazil.

Approximately 80% of individuals infected by DENV, WNV or ZIKV are viremic, and remain asymptomatic throughout the infection, i.e., presenting no symptoms or clinical signs of infection. During the presymptomatic phase or throughout asymptomatic infection, infected individuals may feel well enough to go through a blood donor selection interview without being deferred based on clinical signs. Early symptomatic infections by DENV, WNV and ZIKV exhibit similar symptoms, such as fever, myalgia, arthralgia and headache; however, the infection with each virus can progress to unique clinical manifestations that can be severe or even fatal.

Severe DENV infection (SD) affects the circulatory system, causing increased vascular permeability (“plasma leakage”), hemorrhagic manifestations and decreased platelet levels^12,13^. SD infection currently has no specific treatment, requires hospitalization and life support for survival, and has an increased fatality rate.

WNV infection can cause severe neuroinvasive diseases, such as meningitis, encephalitis and poliomyelitis; symptoms include high fever, headache, neck stiffness, stupor, disorientation, coma, tremors, convulsions, muscle weakness, vision loss, numbness and paralysis^14^.

Zika infection’s clinical manifestations include singular characteristics due to its ability to cross the placental barrier, causing such severe outcomes as congenital syndromes^15^ regardless of asymptomatic presentations^16^. Severe neurological syndromes, including Guillain-Barré syndrome, can also be caused by ZIKV infection^17^.

Factors that trigger clinical manifestations observed in these flavivirus infections, and subsequent development of severe disease, have not been defined and still need to be investigated. Successful identification of biomarkers that differentiate early infection before seroconversion or predict the course of disease may help guide medical interventions, facilitating decisions regarding hospitalization of patients with potential severe disease, thus increasing their chances of survival.

Many aspects of asymptomatic or presymptomatic infections are unknown or poorly understood. Host immune response is a factor associated with severe illness, and the study of immune modulators during presymptomatic/asymptomatic infection may indicate markers involved in the immunopathological response, and thus assist in the identification of patient groups at risk for developing severe disease. In this study, we evaluated soluble marker profiles in plasma specimens from the presymptomatic/asymptomatic acute viremic phase of DENV, WNV and ZIKV infections. Profiling of immune markers may help in the identification of host immune responses to these infections in humans.

## Results

### Flaviviruses display high levels of immune markers with differential profiles associated with each virus

To characterize the immune profiles associated with the acute phase of these flavivirus infections, different sets of soluble markers (a total of 45 molecules) were selected based on literature reports for clinical cases of DENV, WNV and ZIKV. Plasma samples collected from presymptomatic/asymptomatic blood donors infected with either DENV (A-DENV), WNV (A-WNV) or ZIKV (A-ZIKV), and those from non-infected (NI) blood donors (negative control group) were interrogated. All blood collections were performed when donors were apparently healthy enough to be considered eligible for blood donation. The soluble markers included 14 cytokines, eight chemokines, five adhesion molecules, three growth factors, 11 apoptosis markers and four soluble receptors.

**Figure 1:**
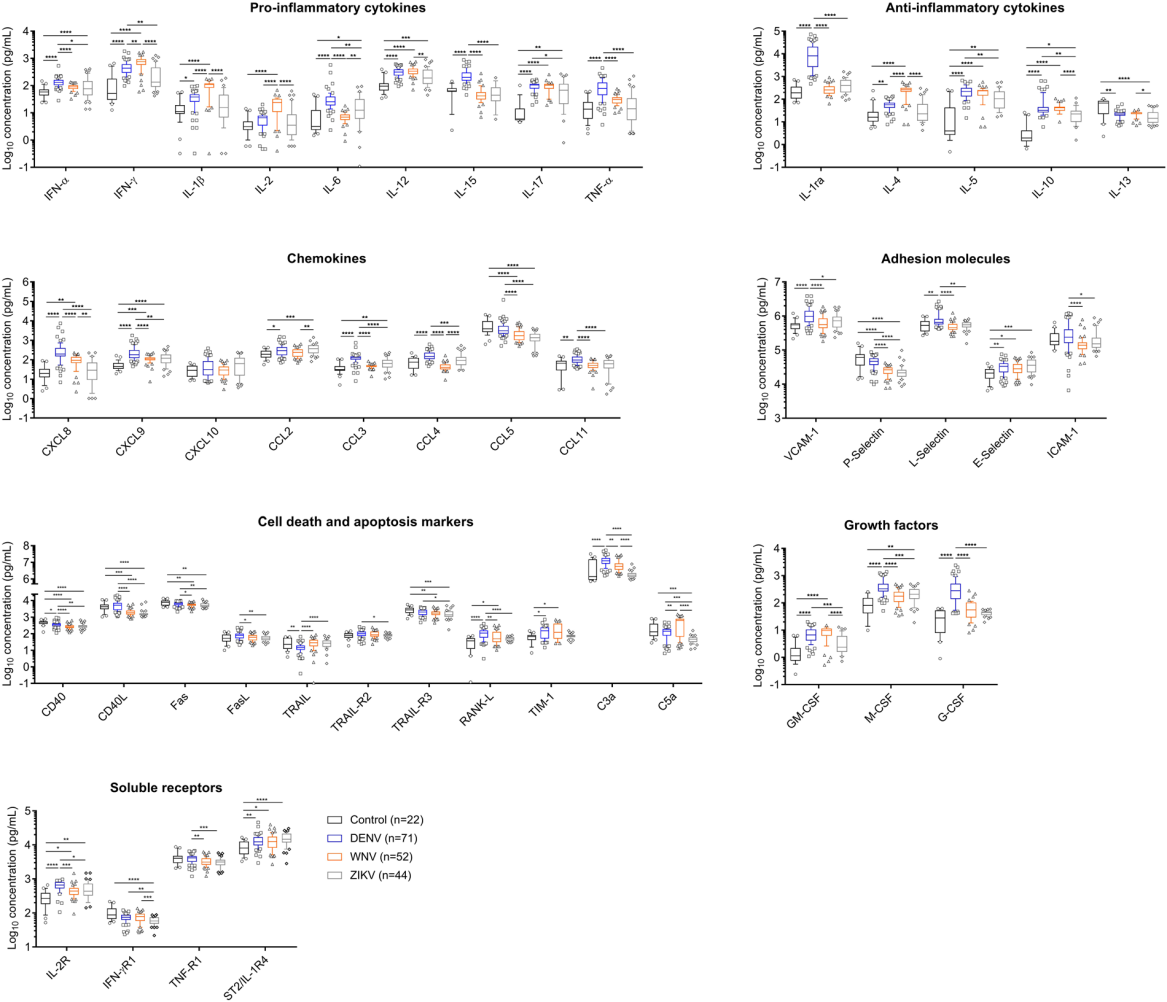
Levels of soluble markers in plasma samples from NI blood donors (Control) (black) and from DENV (blue)-, WNV (orange)- and ZIKV (gray)-infected blood donors. Data are displayed in Log_10_ scale as median with interquartile range of plasma concentration (pg/mL) for each marker. Boxes, median values and interquartile range; whiskers, 10–90 percentile; Points below and above the whiskers are drawn as individual points. Statistical significance was considered at p < 0.05 and is represented by lines and with * for P < 0.05, ** for P < 0.01, and *** for P < 0.001, **** for P < 0.0001.

Samples from infected donors, as compared to NI donors, showed an overall increase in levels of immune markers, even in the absence of symptoms. The A-DENV group displayed elevated levels of 12 cytokines (12/14: interferon alpha (IFN-α), interferon gamma (IFN-γ), IL-1β, IL-6, IL-12, IL-15, IL-17, tumor necrosis factor alpha (TNF-α), IL-1 receptor antagonist (IL-1ra), IL-4, IL-5 and IL-10), six chemokines (6/8: chemokine (C-X-C motif) ligand 8 (CXCL8/IL-8), CXCL9/MIG, chemokine (C-C motif) ligand 2 (CCL2/MCP-1), CCL3/MIP-1α, CCL4/MIP-1β and CCL11/Eotaxin), three adhesion molecules (3/5: vascular cell adhesion molecule-1 (VCAM-1), L-Selectin and E-selectin), three apoptosis-related molecules (3/11: nuclear factor kappa-B ligand (RANK-L), T-cell immunoglobin domain and mucin domain protein 1 (TIM-1) and complement component C3a), all three tested growth factors (3/3: granulocyte-macrophage colony-stimulating factor (GM-CSF), macrophage colony-stimulating factor (M-CSF) and granulocyte colony-stimulating factor (G-CSF), and 2 soluble receptors (2/4: IL-2 receptor (IL-2R), interleukin-1 receptor-like-1 protein (IL-1RL1) also known as ST2/IL-1R4) (Fig. 1, Supplementary Fig. S1).

The A-WNV group showed increased levels of eight cytokines (8/14: IFN-γ, IL-1β, IL-2, IL-12, IL-17, IL-10, IL-4, and IL-5), two chemokines (2/8: CXCL8 and CXCL9), the adhesion molecule E-selectin (1/5), two apoptosis-related molecules (2/11: RANK-L and TIM-1), the growth factor GM-CSF (1/3), and two soluble receptors (2/4: IL-2R and ST2/IL-1R4). Interestingly, this group showed decreased levels of four apoptosis-related molecules (4/9: CD40, CD40L, Fas and tumor necrosis factor-related apoptosis-inducing ligand receptor 3 (TRAIL-R3) (Fig. 1, Supplementary Fig. S1).

The A-ZIKV group exhibited elevated levels of six cytokines (6/14: IFN-α, IL-6, IL-12, IL-17, IL-5 and IL-10), three chemokines (3/8: CXCL9, CCL2 and CCL3), adhesion molecule E-Selectin (1/5), the growth factor M-CSF (1/3), and two soluble receptors (2/4: IL-2R and ST2/IL-1R4). This group showed decreased levels of five apoptosis-related molecules (5/11: CD40, CD40L, Fas, TRAIL-R3 and complement component C5a) (Fig. 1, Supplementary Fig. S1). Additionally, A-ZIKV group samples showed reduced levels of the IFN-γ receptor in comparison with all other tested samples.

Asymptomatic infection with all tested flaviviruses showed similar increased levels of IL-12, IL-17, IL-5, IL-10, CXCL9, E-Selectin and ST2/IL-1R4; and decreased levels of IL-13 and CD40 when compared to the NI control group, suggesting that these markers may represent a common signature for DENV, WNV and ZIKV infections (Fig. 1, Supplementary Fig. S1).

**Figure 2:**
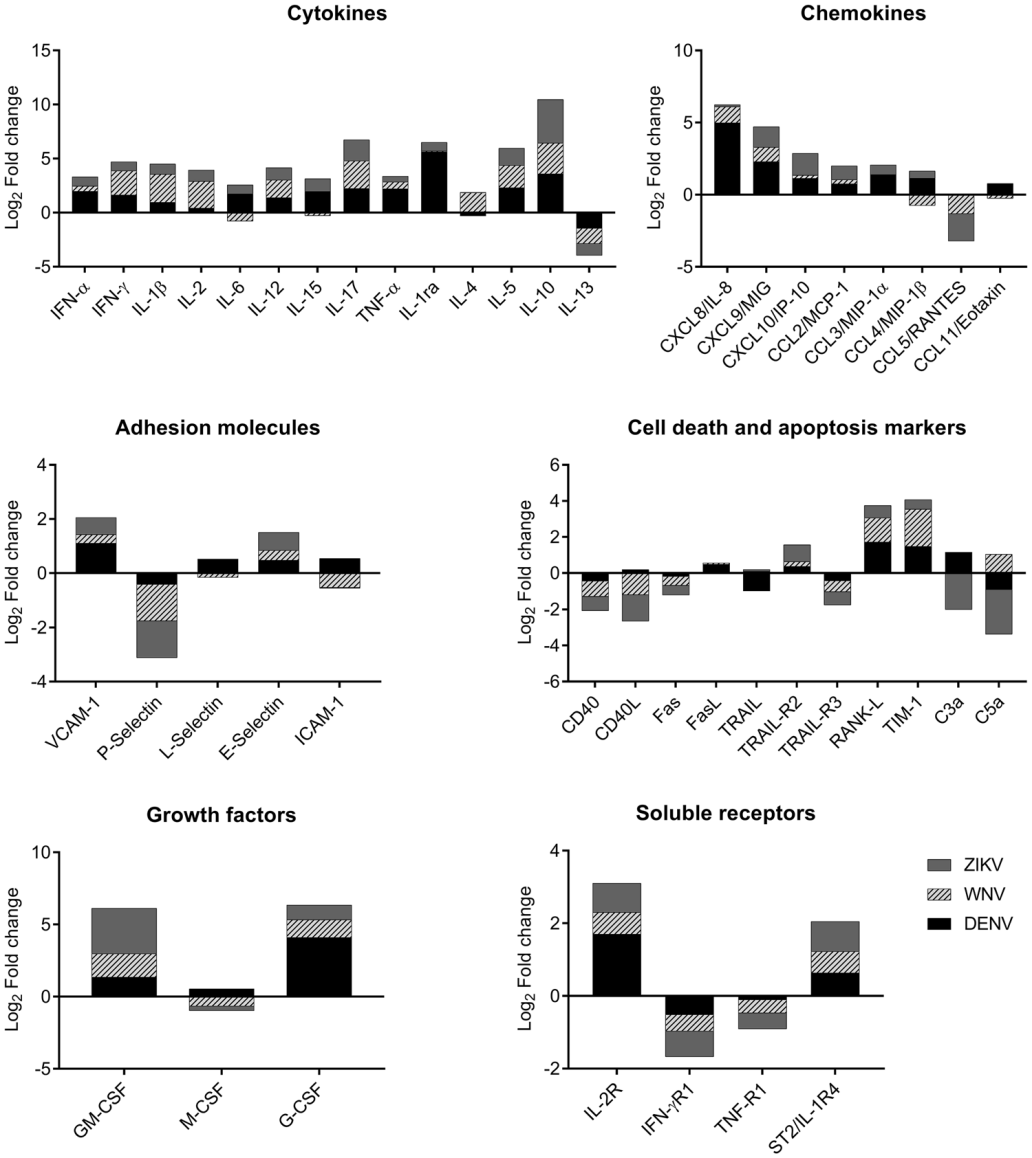
Immunomodulation during viremic flavivirus infection. The modulation of each parameter was expressed as the fold change of average parameter’s concentrations between the DENV, WNV or ZIKV-infected blood donors and the NI blood donors. Data are displayed as Log2 transformed fold changes.

We calculated fold change of concentration average of immune markers between A-DENV, A-WNV and A-ZIKV and NI control groups for each virus to assess the immunomodulation associated with asymptomatic flavivirus infections (Fig. 2). Most markers (24/45) were upregulated in samples from all infected donors, while IL-13, CCL5, P-selectin, CD40, Fas, TRAIL-R3, and the receptors for IFN-γ and TNF, were downregulated. Nevertheless, each specific flavivirus exhibited different modulation of specific molecules, which were either upregulated or downregulated. The A-DENV group had M-CSF, intercellular adhesion molecule-1 (ICAM-1), CCL11 and CD40L upregulated, while IL-4 and tumor necrosis factor-related apoptosis-inducing ligand (TRAIL) were downregulated. The complement component C5a and the cytokine IL-4 were upregulated only in the A-WNV group, while IL-6, IL-15, L-Selectin and CCL4 were downregulated in this group. The A-ZIKV group had no unique upregulated molecule, and only Fas ligand (FasL) was downregulated (Fig. 2).

### A-DENV group had the highest overall frequency of high producers of immune markers

**Figure 3:**
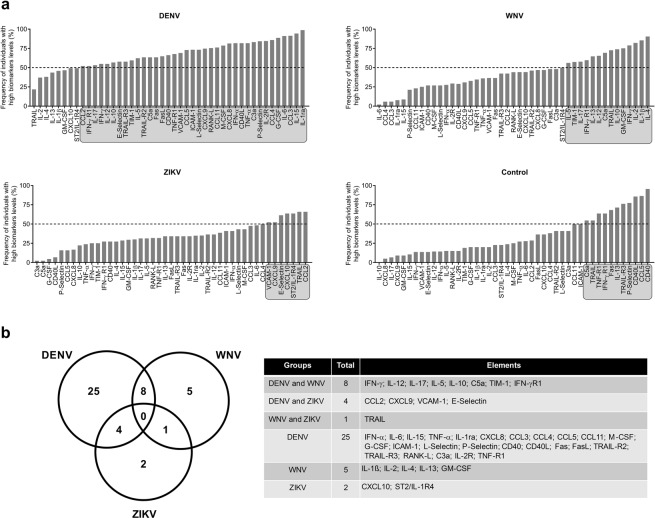
Overall marker signature of soluble markers in plasma samples from DENV-, WNV- or ZIKV-infected blood donors and NI blood donors. (**a**) The marker signatures were assembled as an ascendant curve showing the frequency of individuals with plasma marker levels above the global median for each group. Markers with expression greater than the global median in more than 50% of the individuals are highlighted in gray. (**b**) A Venn diagram was built to characterize the overall frequency pattern of highly-expressed markers in each group. Venn diagram table lists the selected markers that were highly induced in each group.

To further characterize the immunological profiles associated with A-DENV, A-WNV, A-ZIKV and NI groups, individual immune marker signature diagrams were constructed for each group, with the global median value as the cutoff, to categorize each sample as having high or low expression (Supplementary Fig. S2). Ascendant curves of the frequency of plasmas with high marker levels were then built to evaluate changes in the marker’s signature within the groups (Fig. 3a). The results showed highly-induced levels of markers in all groups.

In the A-DENV group, 37 of 45 (82%) markers were highly induced in more than 50% of samples. In A-WNV group 14 of 45 (31%) markers were highly induced in more than 50% of samples. Most of the molecules that were highly induced in the A-DENV group were pro-inflammatory cytokines and chemokines, while the A-WNV group showed an augmented production mainly in pro-inflammatory and anti-inflammatory cytokines. In the A-ZIKV group, only seven of 45 (15.5%) markers (CCL2, CCL4, CXCL9, CXCL10, VCAM-1, E-Selectin and TRAIL) were highly induced in more than 50% of samples. The control group presented a low frequency of individuals with high marker levels for most of the molecules, except for apoptosis-related molecules CD40, CD40L, TRAIL, TRAIL-R3, Fas, TNF-receptor-1 (TNF-R1), C5a, P-Selectin, interferon gamma receptor-1 (IFN-γR1), IL-13 and CCL5 (Figs. 3a, Supplementary Fig. S2).

We built a Venn diagram to summarize the findings observed in the ascendant curves (Fig. 3b); noting that 25 of 45 markers were highly induced only in samples from the A-DENV group. Five of 45 markers were highly induced in samples from the A-WNV group and two of 45 markers were highly induced only in samples from the A-ZIKV group (Fig. 3b). Overall, our findings indicate that DENV elicited a more inflammatory response than did WNV and ZIKV in presymptomatic/asymptomatic infections.

### Asymptomatic flavivirus infections (A-DENV, A-WNV and A-ZIKV) display distinct marker networks

**Figure 4:**
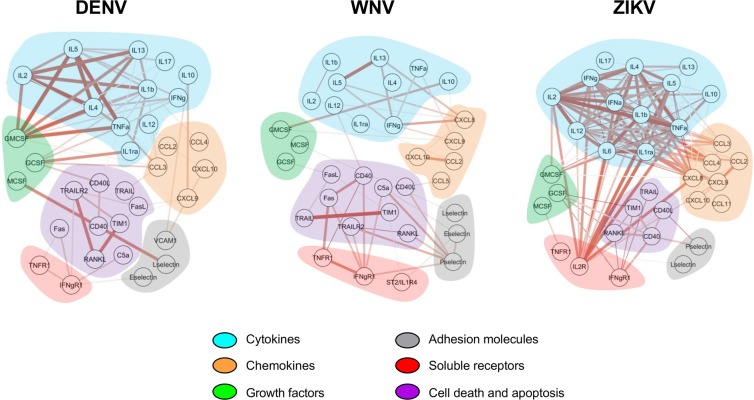
Immune marker networks assembled using Cytoscape software based on Spearman’s correlation matrices and marker functions. Nodes represent the immune marker, and the connection edges represent significant correlation (Spearman >0.5; *p*-value < 0.05). Thicker lines with darker colors indicate higher absolute correlation values. Shaded areas are colored based on marker functions (Blue: cytokines; orange: chemokines; purple: cell death and apoptosis- related molecules; gray: adhesion molecules; green: growth factors; and red: soluble receptors).

Molecule networks were assembled using Cytoscape (version 3.6.1) software and are depicted in Fig. 4. Based on Spearman’s values, the correlations were classified as moderate (0.5–0.7), high (0.7–0.9) and very high (0.9–1.0) positive correlations. Network analysis demonstrated distinct integrative systems between flavivirus-infected donors (Fig. 4). The A-ZIKV group presented with a higher number of very high positive correlations, followed by the A-WNV group, while the A-DENV group presented with only moderate or high positive correlations. The very high positive correlations found in the A-ZIKV group were mainly between cytokine molecules. All negative correlations presented with low Spearman values and were not included in the network analysis.

### Multiple soluble immune markers can be used to distinguish the three flavivirus infections

**Figure 5:**
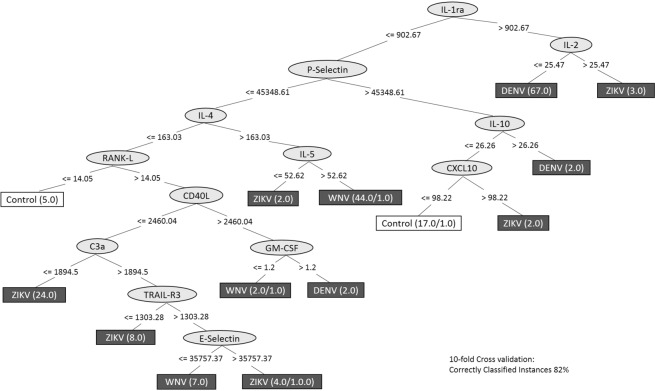
Decision tree analysis to segregate DENV-, WNV- and ZIKV-infected donors from NI blood donors. We used the differentially-expressed markers to construct the dataset and the algorithm C4.5 to build the decision tree using the parameter J48 in Weka (version 3.8.2). The decision tree was generated based on the clustering of all individuals into four groups. The numbers beside the name of each group indicate the values of correct/incorrect register. Ten-fold cross validation values and the frequency of correctly classified instances are shown in the figure.

We performed a decision tree analysis to identify possible biomarkers that could segregate A-DENV, A-ZIKV or A-WNV groups. This analysis indicated that IL-1ra and IL-2 segregated most of the A-DENV samples (94.4%); IL-1ra followed by P-Selectin, IL-4 and IL-5 segregated A-WNV samples (84.6%); and IL-1ra followed by P-Selectin, IL-4, RANK-L, CD40L and C3a segregated 54.5% of A-ZIKV samples. This analysis enabled us to distinguish most of the control group (77%) using IL-1ra, P-Selectin, IL-10 and CXCL10 molecules. It showed a 10-fold cross-validation of 82% (Fig. 5). When we analyzed the AUC (area under the ROC curve), the classifier obtained true positive results in 95.3% (A-DENV), 87.8% (A-WNV), 79.4% (A-ZIKV) and 84.1% (NI) groups. Overall, the decision tree analysis indicated that multiple soluble immune markers could be used to distinguish between these three flavivirus infections.

### Important canonical pathways are shared among DENV, WNV and ZIKV presymptomatic/asymptomatic infections

**Figure 6:**
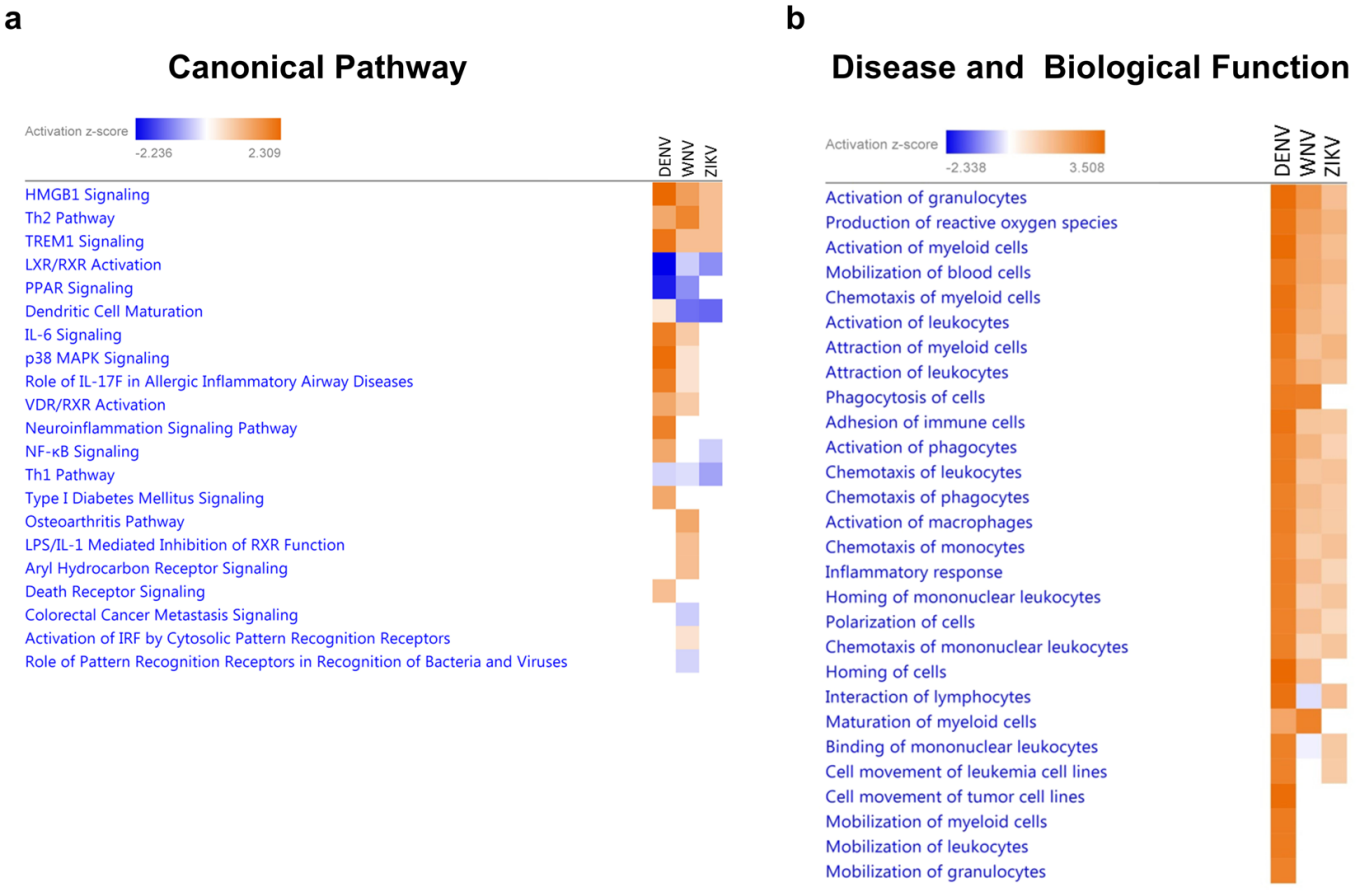
Ingenuity Pathway Analysis predictions in the comparison analysis among DENV-, WNV- and ZIKV-infected donors. Predicted enriched canonical pathways **(a)** and diseases and biological functions **(b)** heatmap analyses were performed using IPA software. The z-score predicts whether a canonical pathway or diseases and biological functions are increased (positive z-score, orange) or decreased (negative z-score, blue) in accordance with the experimental dataset. Darker colors indicate higher absolute z-scores.

Ingenuity Pathway Analysis (IPA) was used to predict enriched canonical pathways and top enriched diseases and biological functions in differentially-expressed immune markers in A-DENV, A-WNV and A-ZIKV plasma samples. The heatmap comparing the 21 most enriched canonical pathways of the infected groups is displayed in Fig. 6a. Several important canonical pathways were shared among the three infected groups. Three of the top five canonical pathways “High mobility group box 1 (HMGB1) signaling”, “T helper 2 (Th2) Pathway” and “Triggering receptor expressed in myeloid cells-1 (TREM-1) Signaling” were activated, while “Liver X receptor/retinoid X receptor (LXR/RXR) Activation” and “Peroxisome proliferator-activated receptor (PPAR) Signaling” were inhibited (Fig. 6a). We observed that the A-DENV group, in comparison to the A-WNV and A-ZIKV groups, showed a higher number of predicted canonical pathways activated.

The absolute z-score was the lowest in the A-ZIKV group for the canonical pathway analysis. Most of the activated canonical pathways were related to activation of cytokines, which corroborated findings that A-DENV plasmas are from high cytokine producers. The heatmap showing the top enriched diseases and biological functions between A-DENV, A-WNV and A-ZIKV are displayed in Fig. 6b. This analysis showed a similar prediction status for all flavivirus-infected groups, including “Activation of granulocytes,” “Production of reactive oxygen species,” “Activation of myeloid cells,” “Mobilization of blood cells” and “Chemotaxis of myeloid cells” (Fig. 6b).

## Discussion

The involvement of immune markers in different clinical phases of *Flavivirus* infections has been widely explored as a means of understanding immunopathogenesis of the diseases. This study compared immune marker levels in plasma samples from blood donors that were reactive for DENV, WNV or ZIKV RNA. Blood donors were presymptomatic/asymptomatic individuals who felt well enough to donate blood. Thus, the study of immune markers in these groups allowed for the investigation of immune-mediated mechanisms contributing to the control of viral infection, as well as for the evaluation of a possible differential profile during presymptomatic/asymptomatic infections. However, the blood samples included in our cohort were from a single time point (time of donation), and no follow-up samples were available for inclusion in the present study. In addition, since no information regarding progression of infection to clinical disease was available we could not correlate the immune marker levels with development of symptoms and/or severity of disease.

Our findings showed that the A-DENV group presented an exacerbated inflammatory response. The A-WNV and A-ZIKV groups showed similar immune profiles in comparison with the NI group. Remarkably, more than 50% of A-DENV samples included in our cohort showed levels of inflammatory cytokines (IFN-α, IFN-γ, IL-1β, IL1-ra, IL-12, TNF-α, IL-6, IL-15 and IL-17) above the global population median, indicating an inflammatory response higher than in the A-WNV and A-ZIKV groups. However, the samples from the A-DENV group were from Puerto Rico, an endemic region for dengue, and these donors probably had been exposed previously to DENV. Most of the ZIKV asymptomatic samples were also collected in Puerto Rico; however, ZIKV did not circulate in that region until late 2015. A possible previous exposure to DENV may be related to differences in the expression pattern observed between the A-DENV and A-ZIKV groups.

Although most (~80%) of DENV-infected individuals did not present with symptoms or clinical signs^12^, progression to SD in symptomatic individuals can be fatal without timely supportive care^3^. Dengue immunopathogenesis has been thought to be mediated by the overproduction and/or an imbalance in cytokine response during the critical phase of the disease, leading to plasma leakage and more severe clinical disease outcomes^18^. It interacts with dendritic cells (DCs), monocytes/macrophages, hepatocytes and endothelial cells, leading to the release of immune mediators during SD^19,20^. Inflammatory cytokines released mainly after T cell activation have been linked to the pathological events triggered by the infection^18,21,22^. SD has been associated with increased production of TNF-α, IFN-γ, IL-1ra, IL-4, IL-6, IL-10, CCL2, CCL3, CCL4, CXCL8 and CXCL10^22–29^. In our study, the A-DENV group also showed increased levels of these molecules, except for CXCL10. In addition to these cytokines and chemokines, increased levels of IFN-α, IL-1β, IL-12, IL-15, IL-17, IL-5, CCL4, CCL11 and CXCL9 were also observed in this group. This high inflammatory response observed in presymptomatic/asymptomatic DENV infection (A-DENV) may represent response to secondary infection since these samples were collected from residents of a DENV-endemic area, whom may have been previously exposed to DENV.

Previous studies have reported elevated levels of IL-12 and CCL4 in patients with mild dengue fever^22,30^. CCL4 is produced by DCs, macrophages and activated natural killer (NK) cells, and is a chemoattractant for NK cells. A correlation between CCL4 plasma levels and NK cells has been observed previously, suggesting an early virus clearance^22^. We observed high levels of IL-12 and CCL4 among the A-DENV group, reinforcing the suggested protective role of IL-12 and CCL4 in DENV infection.

Increased plasmatic levels of cytokines (IFN-α, IFN-γ, TNF-α, IL-4 and IL-10) and chemokines (CCL2, CXCL9 and CXC10) have been seen in samples from WNV-infected blood donors (here referred as A-WNV)^31^. We also observed that the A-WNV group’s profile is characterized by increased levels of pro- and anti-inflammatory cytokines, including IL-2, IFN-γ, IL-12, IL-17, IL-4, IL-5, IL-10 and CXCL9, suggesting a strong and sustained T cell response to control virus replication in presymptomatic/asymptomatic infection. IL-1β signaling through the NLRP3 inflammasome pathway has also been associated with viral control in WNV infection^32^. Additionally, we observed increased IL-1β levels in plasma samples of the A-WNV group, which may indicate an activation of the innate immune response against WNV. Comparison of cytokine levels in serum samples from subjects with a documented history of WNV infection revealed that levels of IL-1β, IL-2, and IL-4 were significantly higher in asymptomatic versus symptomatic infections^33^, corroborating our findings.

Since the reemergence of ZIKV in 2015, several aspects of ZIKV biology and immunopathogenesis have been explored further, but few studies have examined immune signatures during ZIKV disease^34–36^. Previous studies with symptomatic ZIKV-infected patients have shown a different profile of highly- expressed cytokines and chemokines^34,35^. In our study, levels of several immune markers, such as IFN-α, IL-12, IL-6, IL-17, IL-10, IL-5, IL-2R, ST2/IL-1R4, CCL2, CCL3, CXCL9, M-CSF and E-Selectin, were significantly higher in presymptomatic/asymptomatic ZIKV infection (A-ZIKV group) when compared to the NI control group. Although most of these molecules were not highly expressed in the bulk of samples from the A-ZIKV group, correlation among the molecules was remarkably strong, suggesting a balance in the immune response. It is worth noting that the studied population was most likely naïve to ZIKV.

We observed the same pattern of expression for nine of 45 markers for all A-DENV, A-WNV and A-ZIKV groups. Increased levels of IL-12, IL-17, IL-10, IL-5, CXCL9, E-Selectin and ST2/IL-1R4, along with decreased levels of IL-13 and CD40, were found in DENV-, WNV- and ZIKV-asymptomatic groups, compared to the NI control group. IL-17 is known for its role in infection, inflammation and autoimmune diseases^37^. In our study, IL-17 was upregulated during presymptomatic/asymptomatic DENV, WNV and ZIKV infections when compared to non-infected controls. IL-17A has been shown to help WNV clearance by inducing the expression of cytotoxic-mediator genes and promoting CD8^+^ T cell cytotoxicity^38^. The role of IL-17 in ZIKV and DENV infections is not clear. Nevertheless, increased levels of IL-17 were observed in symptomatic ZIKV-infected patients compared to controls^36^, while increased expression of IL-17 was not associated with severity of dengue^20^.

Apoptosis, or programmed cell death, is a series of morphological cell changes, such as nuclear condensation and fragmentation, as well as plasma membrane blebbing, leading to the destruction of the cell. Apoptosis occurs by activation of extracellular death receptors or by intracellular stimuli. The extracellular death receptors (Fas, TRAIL-R and TNFR1) initiate the pathway by recognizing its ligand (FasL, TRAIL and TNF-α)^39^. Interestingly, four apoptosis-related molecules were downregulated in the A-WNV and A-ZIKV groups (CD40, CD40L, Fas and TRAIL-R3). CD40 and CD40-L belong to the TNF and TNFR superfamily and play an important role in modulating immune responses. Their interaction activates DCs through production of pro-inflammatory cytokines and prevention of apoptosis^40^. TRAIL-R3 is a decoy receptor and may inhibit TRAIL-mediated cell death^41^. Thus, the downregulation of these molecules in WNV and ZIKV presymptomatic/asymptomatic infections may suggest an immune response control for an efficient viral clearance.

We have determined plasma marker signatures for A-DENV, A-WNV, A-ZIKV and the NI control group by assessing the frequency of samples with marker levels above the global median. A-DENV samples were characterized by an active immunological profile in which 37 plasma markers were highly induced in more than 50% of the samples. In contrast, the A-WNV and A-ZIKV groups showed only 14 and seven plasma markers, respectively, that were highly induced in more than 50% of the samples. Interestingly, 98.6% of the A-DENV samples showed plasma levels of IL-1ra above the global median, and 90.4% of the WNV samples showed plasma levels of IL-4 above the global median. These two markers were also important in the decision tree analysis. IL-1ra and IL-2 levels segregated most of the A-DENV samples, while IL1ra, P-Selectin, IL-4 and IL-5 levels segregated most of the A-WNV samples. IL-1ra is an anti-inflammatory cytokine, and its production is associated with a feedback mechanism in response to the production of IL-1β in DENV patients^42^. IL-4 also is an anti-inflammatory cytokine, and elevated levels have been reported in asymptomatic WNV-infected individuals^33^, as in our findings. As demonstrated by all analysis performed in the present study, IL-4 seems to be an important marker in the immunoregulatory process of WNV acute infection, contributing to the control of pro-inflammatory T cell-mediated immune responses.

We have observed an immune balance in ZIKV-asymptomatic infection through all analyses performed. Although there was no significant statistical difference in CXCL10 levels in the A-ZIKV group, it was one of two highly-induced molecules in more than 50% of that group only. Indeed, CXCL10 was previously described as a potential biomarker for acute ZIKV infection^36^, with more prominent expression in the convalescent stage^43^. Together, these results suggest that measuring multiple soluble immune markers could be used to distinguish DENV, WNV and ZIKV presymptomatic/asymptomatic infections.

The IPA analysis further showed that the upregulated pro-inflammatory cytokines in the A-DENV group are involved in the HMGB1 signaling pathway. HMGB1 is a nucleosomal protein that regulates transcription. It is released passively by necrotic cells, but it can also be actively released by immune cells^44,45^, and induces the production of pro-inflammatory cytokines by monocytes, macrophages and DCs^46–48^. The release of HMGB1 also increases the expression of ICAM-1, VCAM-1 and pro-inflammatory cytokines in endothelial cells, suggesting a propagation of the immune response during infection or injury by HMGB1^49^. It has been shown that DENV infection induces HMGB1 release by DCs, which further stimulates the production of cytokines^50^.

The TREM-1 signaling pathway is also involved with the elevated levels of pro-inflammatory cytokines and chemokines found in DENV-infected donors in the IPA analysis. TREM-1 signaling is related to an amplification of the inflammatory response through increased production of pro-inflammatory cytokines and chemokines in bacterial and viral infections^51^.

The restriction to a single time-point of sample collection in this study does not allow correlation to clinical outcome but provides the unique opportunity of investigating level of soluble immune markers in presymptomatic/asymptomatic DENV, WNV or ZIKV infections. The results show a differential expression pattern of soluble immune markers in the presymptomatic/asymptomatic infection as compared to non-infected control subjects with comparable demographic features. Therefore, these findings pave the way to further investigation of immune mediator profiles, which may lead to early identification of individuals at risk for severe disease and assist managing pregnancies at risk in suspected cases of ZIKV infection.

## Materials and Methods

### Study population

This study included 189 anonymized residual plasma samples from blood donors who were asymptomatic and eligible as donors at the time of donation, but screened positive for DENV, WNV or ZIKV viral RNA, indicating that they were in acute viremic phase of infection. Of these, 167 were reactive for DENV (n = 71), WNV (n = 52) or ZIKV (n = 44) RNA, and 22 negative samples from non-infected individuals were used as the negative control group. The 22 non-infected samples were retested in our laboratory, and confirmed negative for DENV, WNV or ZIKV RNA by reverse transcription polymerase chain reaction (RT-PCR). All samples collected in this study were approved by an Institutional Review Board (IRB), which included signed informed consent. All research activities were guided by the ethical principles of respect for persons, beneficence, and justice, in accordance with The Belmont Report. Study protocols were reviewed and approved by the FDA Research in Human Subjects Committee (FDA IRB) as follows: DENV samples under Protocol # 13-001B; WNV samples under Protocol # 03127B; ZIKV samples under Protocol # 17-001B; and negative samples under Protocol # 03-120B.

DENV-positive samples were collected during the epidemic seasons of 2012–2013 in Puerto Rico by the American Red Cross (ARC). Blood donation samples were initially found to be reactive for DENV RNA by an investigational nucleic acid test (NAT) assay (DENV TMA assay, Hologic, Inc., formerly Gen-Probe, Inc., San Diego, CA), in use under an FDA-approved Investigational New Drug Application (IND) to screen blood donations from Puerto Rico for DENV RNA^52^. Residual samples from tubes used for blood screening by the ARC were unlinked and shipped frozen to our laboratory for processing and testing.

WNV-positive plasma samples were obtained from residual blood specimens collected between 2012 and 2013 from blood donors who tested reactive for WNV RNA by FDA-approved commercial NAT assays used to screen blood donations. These samples were from various sources and part of a repository at FDA’s Center for Biologics Evaluation and Research (CBER). For this study, we selected samples from blood donors who lived in areas without reported *Aedes* mosquito activity.

ZIKV-positive plasma samples were collected during the 2016 epidemics in Puerto Rico and Florida. Samples tested reactive for ZIKV RNA using the cobas NAT Zika test (Roche Molecular Systems, Inc., Pleasanton, CA) under an FDA-approved IND to screen blood donations.

Uninfected samples used as the negative control were collected at the National Institute of Health Division of Transfusion Medicine and shipped the same day via courier to the FDA’s laboratory for processing and testing. Plasma samples were aliquoted and stored at −20 °C until testing.

### Luminex-based assays: multiple soluble molecules analysis

The concentration of soluble molecules in plasma samples was measured using Luminex xMAP technology with different bead panels from different companies. Briefly, Luminex-based assays use microspheres (beads) internally labelled with distinct proportions of two fluorophores, which enables the differentiation of one bead from another, and which are pre-coated with analyte-specific antibodies. A fluorescent Streptavidin-phycoerythrin (PE)-conjugated antibody was added for the identification of the molecules’ concentrations, calculated based on the standard curve concentrations. Results are expressed in picograms per milliliter (pg/mL).

The Cytokine Human 25-Plex Panel for Luminex Platform LHC0009 (Life Technologies, Frederick, MD), with polystyrene beads and standard concentration ranging from 4.2 to 0.5 × 10^4^ pg/mL, was used to measure the concentration of GM-CSF, TNF-α, IFN-α, IFN-γ, IL-2, IL-1β, IL-4, IL-5, IL-6, IL-7, IL-10, IL-12 (p40⁄p70), IL-13, IL-15, IL-17, CCL2/MCP-1, CCL3/MIP-1α, CCL4/MIP-1β, CCL5/RANTES, CCL11/Eotaxin, CXCL8/IL-8, CXCL9/MIG, CXCL10/IP-10, IL-2R and IL-1ra. The bead-based multiplex assay for the Luminex platform kit Human Luminex Screening Assay [LXSAH] (R&D Systems, Minneapolis, MN)–with polystyrene beads and a standard concentration ranging from 1.8 × 10^7^ to 1.0 × 10^3^ pg/mL–was used to measure the concentration of CD40, CD40L, C5a, Fas, FasL, TIM-1, TRAIL, TRAIL-R2, TRAIL-R3, RANK-L, M-CSF, G-CSF, ICAM-1, VCAM-1, E-Selectin, L-Selectin, P-Selectin, IL-1RL1 also known as ST2/IL-1R4, TNF-R1 and IFN-γR1.

Subjects’ plasma samples were assayed following the manufacturer’s protocols. Samples and standard curves were run in duplicate. Fluorescence signals were detected using the multiplex array reader Luminex 200™ System (Invitrogen, Grand Island, NY) and Bio-Plex 200 system (Bio-Rad, Hercules, CA) and analyzed using the Bio-Plex manager 6.1 software (Bio-Rad).

Quantitative detection of human C3a, the most abundant protein of the complement systemin plasma samples was performed by enzyme-linked immunosorbent assay (ELISA) (eBioscience, Vienna, Austria) following the manufacturer’s protocols. Samples and standard curves were run in triplicate. Absorbance of each microwell was read on a microplate reader (SpectraMax M5, Molecular Devices, San Jose, CA). The C3a concentration was determined using the SoftmaxPro software (Molecular Devices, San Jose, CA) based on the concentration read from the standard curve.

### Statistical analysis and data mining

Statistical analysis was performed using a non-parametric Mann-Whitney test for two independent groups, and a Kruskal-Wallis test followed by Dunn’s *post hoc* analysis test when comparing three or more groups via GraphPad Prism version 7.0 software (GraphPad, San Diego, CA). Statistical significance was defined as a *p* value of ≤0.05. To assess individual immune marker signatures, we calculated the global median value for each marker using a single data set including all samples (i.e. comprised of A-DENV, A-WNV, A-ZIKV and NI groups) as previously described^53^. The global median values were further used as a cutoff to classify individuals as high or low producers for each marker (those with results above or below the global median value, respectively). The percentage of high producers were shown in format of ascendant marker curves and Venn diagram. Spearman’s test was used to identify associations between the immune markers. Significant correlations (*p*-value < 0.05 and r > 0.5) were assembled in Cytoscape software version 3.6.1 (www.cytoscape.org)^54^ to assess an integrated network of immune markers. To verify immune patterns associated with the flavivirus infection, we performed a decision tree analysis using the algorithm C4.5 in Weka software version 3.8.2 (Waikato Environment for Knowledge Analysis, University of Waikato, Hamilton, New Zealand)^55^ as well as a 10-fold cross validation to assess the predictive quality of the model.

Canonical pathways and enriched diseases and/or biological functions analysis were performed by Ingenuity Pathway Analysis (IPA) software (QIAGEN Bioinformatics, Redwood City, CA). Differentially-expressed immune markers from asymptomatic DENV, WNV and ZIKV infected donors were uploaded in IPA. Fisher’s exact test was used to calculate a *p*-value determining the probability that each canonical pathway and biological function and/or disease assigned to these data sets were due to chance alone.

## Supplementary information

Supplementary material

## Data Availability

The datasets generated during and/or analysed during the current study are available from the corresponding author on reasonable request.
