# Supplementary material for: Differential Pattern of Soluble Immune Markers in Asymptomatic Dengue, West Nile and Zika Virus Infections
Source: Sci Rep. 2019 Nov 20;9:17172. doi: 10.1038/s41598-019-53645-w (PMC6868147; doi:10.1038/s41598-019-53645-w)
Supplement: Supplementary file 1 — Supplementary material [file 41598_2019_53645_MOESM1_ESM.pdf]

Differential Pattern of Soluble Immune Markers in Asymptomatic Dengue, West Nile and Zika Virus Infections

Rafaelle Fares-Gusmao[1§], Bruno Coelho Rocha[1§], Emilia Sippert[1], Marion Lanteri[2,3], Germán Áñez[1,#], Maria Rios[1*]

[1]Office of Blood Research and Review (OBRR), Center for Biologics Evaluation and Research (CBER), U.S. Food and Drug Administration (FDA), Silver Spring, MD, USA.

[2]Blood Systems Research Institute, San Francisco, CA, USA.

[3]Department of Laboratory Medicine, University of California San Francisco, San Francisco, CA, USA.

[*]**Correspondence:**
Dr. Maria Rios
maria.rios@fda.hhs.gov

[§]Authors have contributed equally to this paper

#Present address: Sanofi Pasteur, Swiftwater, PA, USA

Supplementary Information

Supplementary Figure S1. Levels of soluble markers in plasma samples from NI blood donors and from DENV-, WNV- and ZIKV-infected blood donors. Data are displayed as median with interquartile range of plasma concentration (pg/mL) for each marker. Statistical significance was at $p<0.05$ and is represented by dashed lines.

Supplementary Figure S2. Overall marker signature of soluble markers in plasma samples from DENV-, WNV- and ZIKV-infected blood donors and NI blood donors. The diagrams were constructed using the global median value as the cutoff point to identify individuals as low or high producers in each group. Individuals with plasma levels above the global median are colored gray and the relevant frequencies (≥ 50%) of individuals with high serum levels are depicted in bold and underline format.

Figure S1

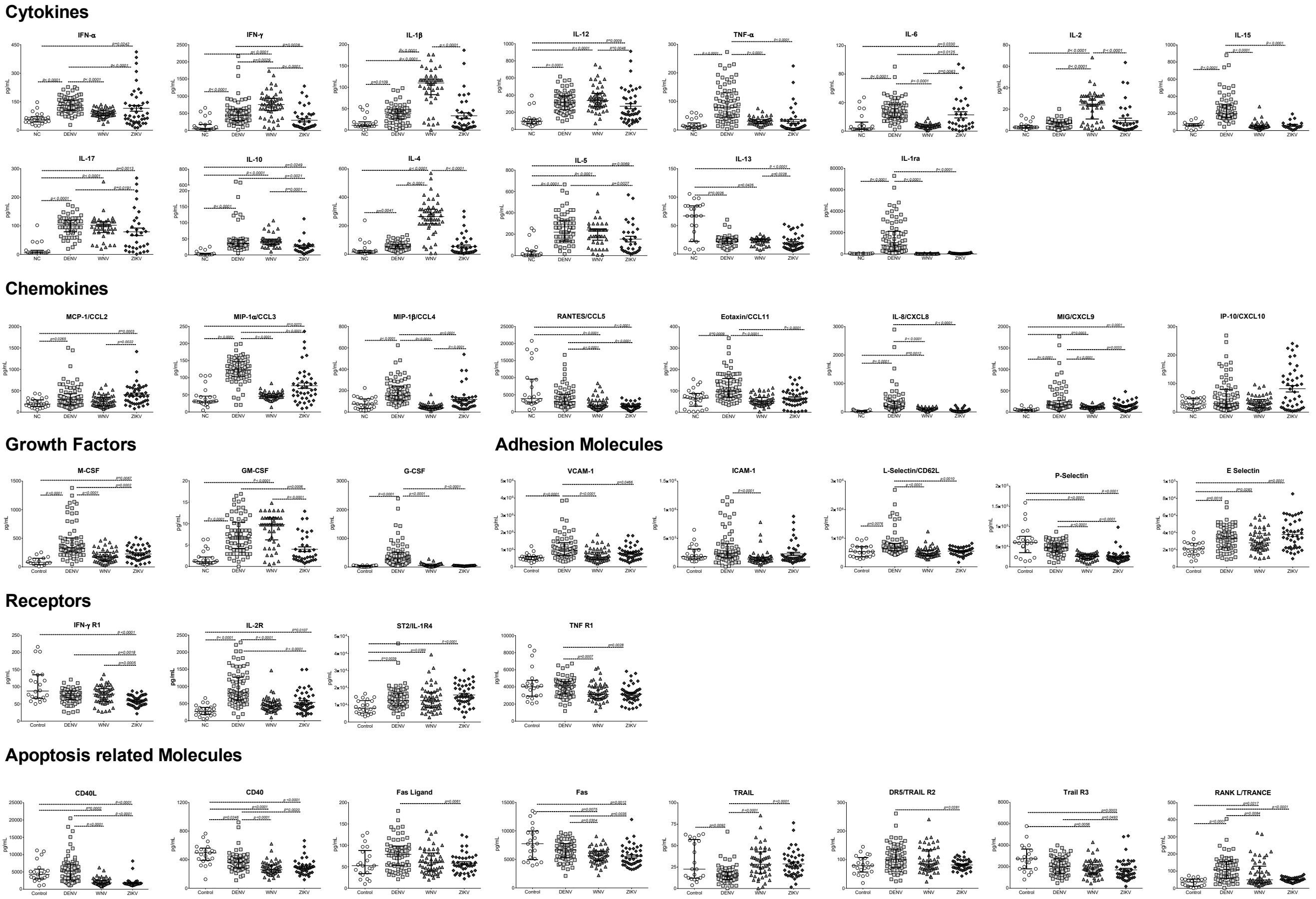

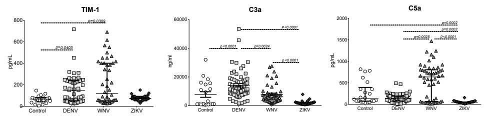

Figure S1. Levels of soluble immune markers in plasma samples from NI blood donors and from DENV-, WNV- and ZIKV-infected blood donors. Data are displayed as median with interquartile range of plasma concentration (pg/ml) for each marker. Statistical significance was considered at p<0.05 and are represented by dashed lines.

Figure S2

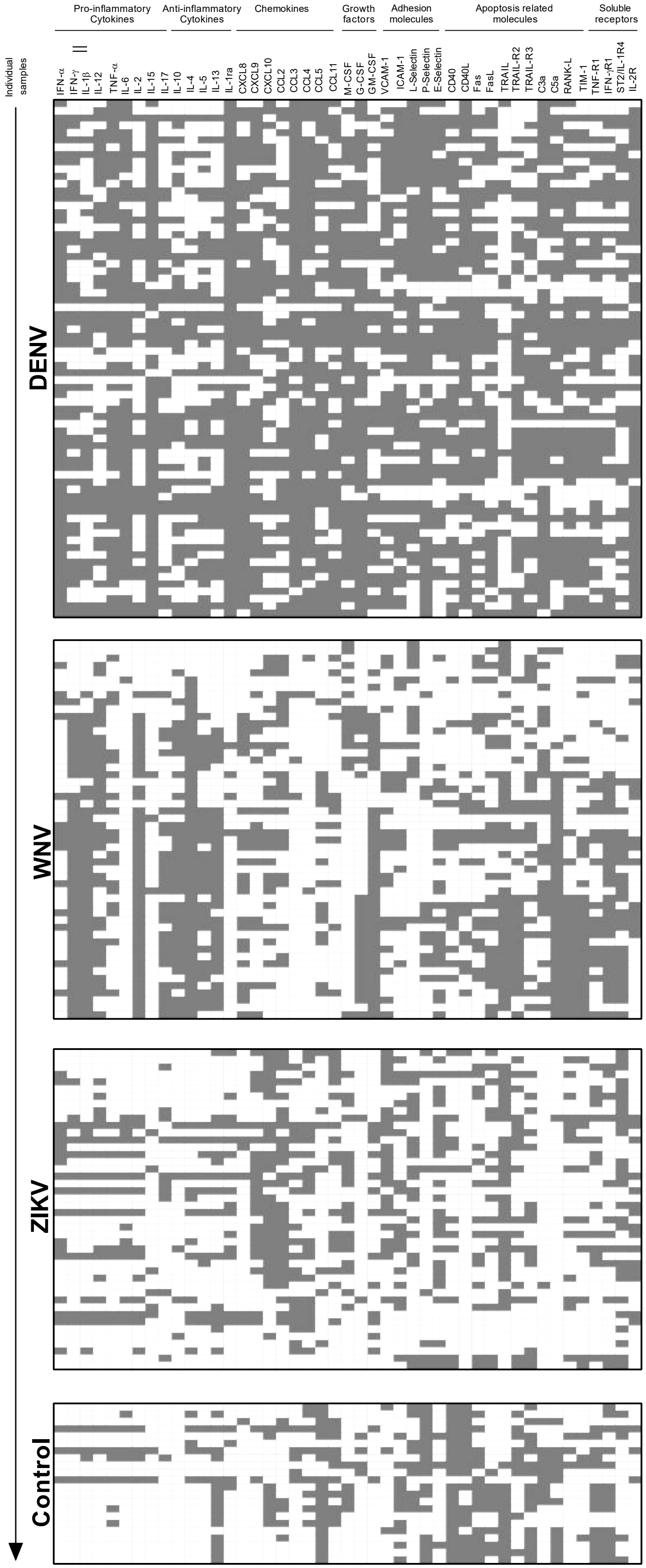

Figure S2. Overall biomarker signature of soluble immune markers in plasma samples from DENV-, WNV-, ZIKV-infected blood donors and NI blood donors. The diagrams were constructed using the global median value as the cut-off point to identify each individual as a low or high producer in each group. Individuals with plasma levels above the global median are colored in gray and the relevant frequency (≥ 50%) of individuals with high serum levels are depicted in bold and underline format.
